# Prenatal diagnosis of 6pter-p24 deletion syndrome in a fetus from a Han Chinese family: A case report

**DOI:** 10.1097/MD.0000000000019246

**Published:** 2020-02-21

**Authors:** Qing-yang Shi, Yan-hong Liu, Yong-sheng Zhang, Xiao-wei Yu

**Affiliations:** Center for Reproductive Medicine and Center for Prenatal Diagnosis First Hospital, Jilin University.

**Keywords:** 6pter-p24 deletion syndrome, Chromosome, magnetic resonance imaging, prenatal diagnosis, prenatal ultrasound

## Abstract

**Introduction:**

Chromosome 6pter-p24 deletion syndrome (OMIM #612582) is a rare genetic disorder characterized by deletion of the distal part of 6p. Human 6p deletion syndromes result in a variety of congential malformations.

**Patient concerns:**

The fetus was the fourth child born to healthy non-consanguineous parents with no relevant family history.

**Diagnosis:**

The fetus was diagnosed with 6pter-p24 deletion syndrome through prenatal ultrasound, magnetic resonance imaging, and chromosomal microarray analysis. The fetus had brain, skeletal, and heart malformations. The fetus was cytogenetically normal. Chromosomal microarray analysis detected an interstitial 7.999Mb deletion within the 6p25.1p24.3 region of chromosome 6.

**Interventions:**

There was no treatment for the fetus.

**Outcomes:**

Pregnancy was terminated.

**Conclusions:**

To the author's knowledge, the present case is one of the first to report the prenatal diagnosis of 6pter-p24 deletion syndrome in a fetus. No published reports have described the diagnosis of 6pter-p24 deletion syndrome using multiple technologies during the antenatal period; therefore, our findings may provide a reference for other clinicians. The clinical features and pathophysiology of this prenatal diagnosis are discussed.

## 1 Introduction

Chromosome 6pter-p24 deletion syndrome (OMIM #612582) is a rare genetic disorder characterized by deletion of the distal part of 6p. Chromosome 6pter-p24 deletion syndrome manifests as developmental delay/mental retardation, reduced muscle tone, Dandy-Walker malformation, hearing, eye, and cardiac abnormalities, and abnormal skull shape.^[1]^ According to Unique, a rare chromosome disorder support group, in 2017, there were 74 reported cases of isolated 6p deletions, 44 reported cases of 6p deletions associated with deletions and duplications on other chromosomes, and 32 reported cases of 6p25 deletions involving no other chromosome change.^[2]^ Here, we report the case of a fetus diagnosed with terminal 6p deletion syndrome through prenatal ultrasound, magnetic resonance imaging (MRI), and chromosomal microarray analysis. The clinical features and pathophysiology of this prenatal diagnosis are discussed.

## 2 Case report

### 2.1 Medical history

The fetus was the fourth child born to healthy non-consanguineous parents with no relevant family history. Previously, the mother had experienced a twin pregnancy that was terminated at 26 + 2 weeks in the Third Hospital of Beijing University. The pregnancy was complicated by hypertension, severe preeclampsia, twin to twin transfusion syndrome, and selective intrauterine growth restriction. The growth-restricted twin died at 20 + 6 weeks and had a unilateral club foot, hypomyotonia, and pericardial effusion. The co-twin also died. The third child was born a healthy boy.

### 2.2 Antenatal ultrasound scan

**Figure 1:**
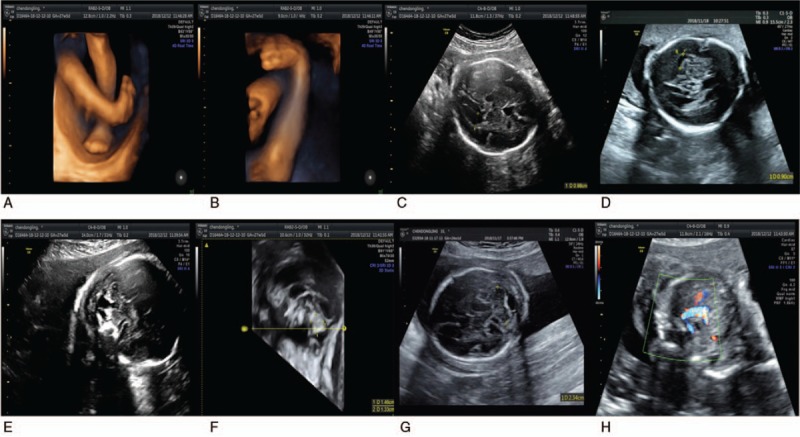
Fetal ultrasound A, B: Right clubfoot; C, D: abnormally enlarged brain ventricles: left 0.98 cm, right 0.90 cm; E, F, G: an enlarged cisterna magna: 0.65 cm, anterior-posterior length of the cerebellar vermis, height of the cerebellar vermis, transcerebellar diameter; H: aberrant right subclavicular artery, mild tricuspid reflux.

Regarding the fourth child, an antenatal ultrasound scan at 23 + 6 weeks revealed the fetus had brain (abnormally enlarged brain ventricles: left 0.98 cm, right 0.90 cm, an enlarged cisterna magna: 0.65 cm, anterior-posterior length of the cerebellar vermis: 1.33 cm, height of the cerebellar vermis: 1.46 cm, area of the cerebellar vermis: 1.69cm2, circumference of the cerebellar vermis: 5.26 cm; all less than the average reference value for that gestational week), skeletal (unilateral clubfoot), and heart (aberrant right subclavicular artery, mild tricuspid reflux) malformations (Fig. 1).

### 2.3 Fetal MRI (second affiliated hospital of China medical university)

Fetal MRI showed the sulcus, gyrus, and convolutions of the cerebrum were normal, the anterior-posterior length of the cerebellar vermis and the height of the cerebellar vermis were less than the average reference value for that gestational week.

At this time, the parents underwent genetic counseling and opted for amniocentesis.

### 2.4 Cytogenetic analysis

Peripheral blood (0.5 mL) collected from the parents into sterile tubes containing 30 U/mL heparin. Lymphocytes were isolated and cultured in sterile tubes with Yishengjun culture media (Yishengjun; Guangzhou Baidi Biotech, Guangzhou, China) for 72 h and treated with 20 μg/mL colcemid for 1 h. G-banding of metaphase chromosomes and karyotype analysis were performed. Images were captured using Leica CW-4000 software (Leica, Wetzlar, Germany). Karyotypes were described in accordance with the International System for Human Cytogenetic Nomenclature (ISCN) 2013. A total of 20 metaphases were counted, of which 6 were analyzed. the parents and the third child were cytogenetically normal.

### 2.5 Cytogenetic analysis for detection of aneuploidy

**Figure 2:**
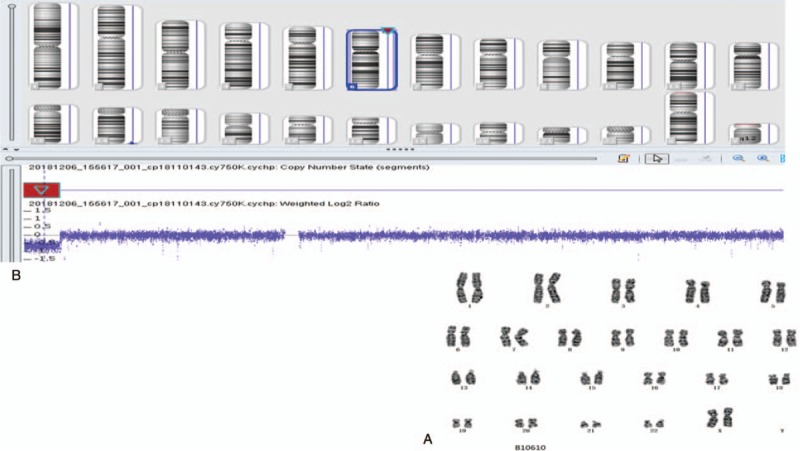
Cytogenetic and chromosomal microarray analyses A: the fetus was cytogenetically normal; B: chromosomal microarray analysis detected an interstitial deletion within 6p25.1p24.3.

Chromosomal analyses of amniocentesis samples were carried out using standard protocols. Amniotic fluid samples were cultured in AmnioMAX-C100 culture medium (Invitrogen, Carlsbad, CA). Metaphase chromosomes were stained using the Giemsa-trypsin-Giemsa banding method. Twenty metaphases were analyzed, and karyotypes were described in accordance with ISCN 2013. The fetus was cytogenetically normal. The chromosomes of the parents and brother were normal (Fig. 2).

### 2.6 Chromosomal microarray

Chromosomal microarray analysis was performed using the CytoScan 750K array (Affymetrix, Santa Clara, CA) according to the manufacturer's protocol. 250 ng of fetal genomic DNA were labeled, and the microarrays were processed in the Affymetrix GeneChip Fluidics Station 450. Affymetrix image files (.CEL file) were analyzed using Analysis Suite v3.3 Software (Affymetrix). The February 2009 human reference sequence (GRCh37/Hg19) produced by the Genome Reference Consortium was used to interpret genomic data. Chromosomal microarray analysis detected an interstitial 7.999Mb deletion within the 6p25.1p24.3 region of chromosome 6 (Fig. 2).

Finally, the diagnosis was made at 27+6 weeks, the parents of the fetus decided to terminate pregnancy with no treatment.

## 3 Discussion and conclusions

Human 6p deletion syndromes result in a variety of congential malformations. This case study describes a fetus diagnosed with terminal 6p deletion syndrome through prenatal ultrasound, MRI, and chromosomal microarray analysis. 6p-associated phenotypes can arise from two distinct deletion syndromes. Terminal deletions with breakpoints within the 6p24-pter region are associated with craniofacial defects such as hypertelorism, high arched palate, and cleft lip or palate, structural eye abnormalities such as posterior embryotoxon, hearing loss, heart defects such as supraventricular tachycardia, atrial, venatrial and ventricular septal defects and patent ductus arteriosus and foramen ovale, skeletal malformations, and brain anomalies such as Dandy-Walker malformation, hydrocephalus, polymicrogyria, and hypoplasia of the corpus callosum, cerebellum and brainstem.^[3,4]^ Interstitial deletions of the 6p24 to 6p22 region are associated with a short neck, craniofacial defects, structural eye anomalies, cardiac, kidney, and limb defects, and developmental delay.

Molecular characterization of the deletions can identify genes that are responsible for these phenotypes. Phenotypic manifestations of 6p deletions are usually apparent at birth, implicating these genes in embryonic and fetal development. The role of most genes (39 OMIM genes) in the 6p25 region is unclear. In the present case study, chromosomal microarray analysis detected an interstitial deletion within 6p25.1p24.3. Fetal ultrasound showed abnormally enlarged brain ventricles and an enlarged cisterna magna, a unilateral clubfoot, an aberrant right subclavicular artery, and mild tricuspid reflux. MRI images confirmed the presence of brain anomalies. To the author's knowledge, the present case is among the first to report MRI images of a fetus diagnosed with terminal 6p deletion syndrome.^[3,4]^

Forkhead box C1 (FOXC1), found in band 6p25.3 between base pairs 1,610,680 and 1,614,131, is required for the development of mesenchyme-derived tissues such as somites, heart, bone, and brain. FOXC1 encodes a forkhead box transcription factor. Previous studies have shown glaucoma, iris hypoplasia, Axenfeld-Rieger syndrome, and Dandy-Walker malformation are associated with mutations in FOXC1.^[5]^ Cerebellum abnormalities, including mega cisterna magna or cerebellar vermis hypoplasia, occur in FOXC1 null mice and in patients with mental retardation and known FOXC1 gene deletions or missense mutations. Cerebellar malformations are more severe in individuals with intragenic FOXC1 point mutations and small deletions or duplications in the nearby FOXF2 or GMDS genes. Malformations of the spine or vertebrae and often the hands and feet have also been reported in patients with FOXC1 deletions. It is possible that deletion of FOXC1 contributed to the phenotype of the fetus in this case study; however, the parents refused further analyses that would have allowed molecular identification of the genes responsible for the fetal phenotype.

In summary, we report the case of a fetus diagnosed with 6pter-p24 deletion syndrome through prenatal ultrasound, MRI, and chromosomal microarray analysis. The fetus had brain, skeletal, and heart malformations. The fetus was cytogenetically normal; however, chromosomal rearrangements smaller than 10-Mb could not be detected using our methodology. Chromosomal microarray analysis detected an interstitial 7.999Mb deletion within the 6p25.1p24.3 region of chromosome 6. To the author's knowledge, the present case is among the first to report MRI images of a fetus diagnosed 6pter-p24 deletion syndrome. No published reports have described the diagnosis of 6pter-p24 deletion syndrome using multiple technologies during the antenatal period; therefore, the findings may provide a reference for other clinicians. The parents refused further analyses that would have allowed molecular identification of the genes responsible for the fetal phenotype.

## Author contributions

**Conceptualization:** Qing-yang Shi, Yan-hong Liu, Yong-sheng Zhang, Xiao-wei Yu.

**Data curation:** Qing-yang Shi, Yan-hong Liu, Yong-sheng Zhang, Xiao-wei Yu.

**Formal analysis:** Qing-yang Shi.

**Funding acquisition:** Qing-yang Shi.

**Project administration:** Yong-sheng Zhang.

**Supervision:** Yan-hong Liu.

**Validation:** Qing-yang Shi, Yan-hong Liu.

**Visualization:** Qing-yang Shi, Xiao-wei Yu.

**Writing – original draft:** Xiao-wei Yu.
